# Supplementary material for: Dissecting Total Plasma and Protein-Specific Glycosylation Profiles in Congenital Disorders of Glycosylation
Source: Int J Mol Sci. 2020 Oct 15;21(20):7635. doi: 10.3390/ijms21207635 (PMC7589176; doi:10.3390/ijms21207635)
Supplement: Supplementary file 1 [file ijms-21-07635-s001.zip › CDG_IJMSci_Supplemental.pdf]

Supporting Material Figures

Dissecting total plasma and protein-specific glycosylation profiles in congenital disorders of glycosylation

Agnes L. Hipgrave Ederveen[1], Noortje de Haan[1], Melissa Baerenfaenger[3], Dirk J. Lefeber[2,3,*] and Manfred Wuhrer[1,*]

1 Center for Proteomics and Metabolomics, Leiden University Medical Center, Albinusdreef 2, 2333 ZA Leiden, The Netherlands;
2 Department of Laboratory Medicine, Translational Metabolic Laboratory, Radboud University Medical Center, Geert Grooteplein 10, Nijmegen, 6525 GA, The Netherlands;
3 Department of Neurology, Donders Institute for Brain, Cognition and Behavior, Radboud University Medical Centre, Geert Grooteplein 10, Nijmegen, 6525 GA, The Netherlands;
* Correspondence: Dirk.Lefeber@radboudumc.nl ; m.wuhrer@lumc.nl ; Tel.: +31-24-30-93487 (D.J.L.); +31-71-62-66989 (M.W.)

Table of Contents

Figure S1: Glycoforms of immunoglobulin G (IgG) detected by MALDI-TOF-MS (Page S2)

Figure S2: Total plasma N-glycome (TPNG) fucosylation (Page S3)

Figure S3: Sialylation per galactose (A2GS) on IgG (Page S4)

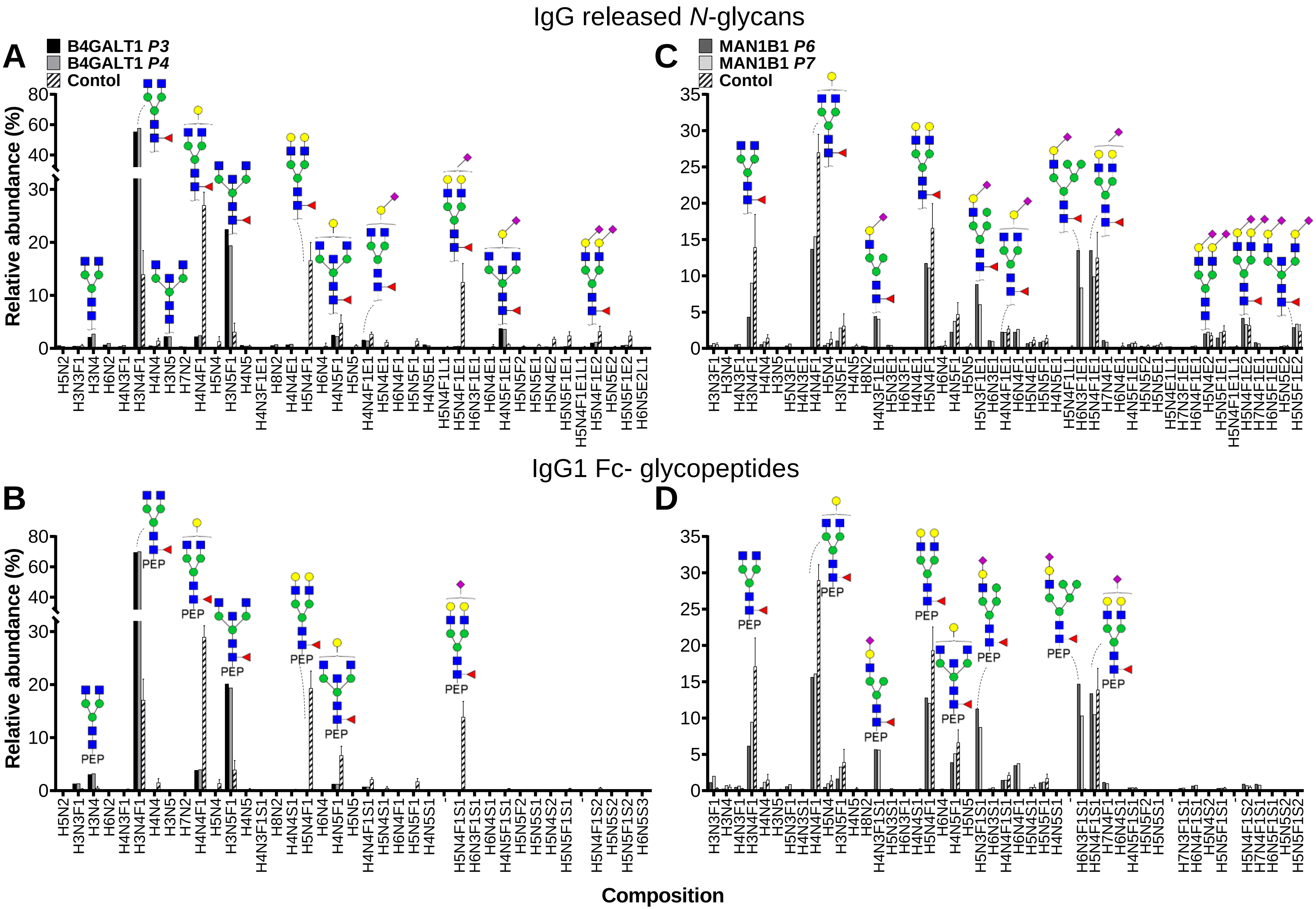

Supplementary figure S1. Glycoforms of immunoglobulin G (IgG) detected by time-of-flight (MALDI-TOF)-MS of released *N*-glycans after linkage-specific sialic acid derivatization and nanoliquid chromatography mass spectrometry (nanoLC-MS) of IgG1 Fc-*N*-glycopeptides. The relative abundance of released *N*-glycans from total IgG of patients with B4GALT1-CDG (**A**) and MAN1B1-CDG (**C**) and IgG1 Fc-*N*-glycopeptides of B4GALT1-CDG (**B**) and MAN1B1-CDG (**D**) patients compared to healthy controls (*n* = 10), bar showing median and standard deviation. Except for the sialic acid linkage in the MALDI-TOF-MS analysis (**A** and **C**) the monosaccharide linkages were not determined. The proposed glycan structures are based on fragmentation and literature. *N*-glycan annotation and abbreviations are described in Figure 1.

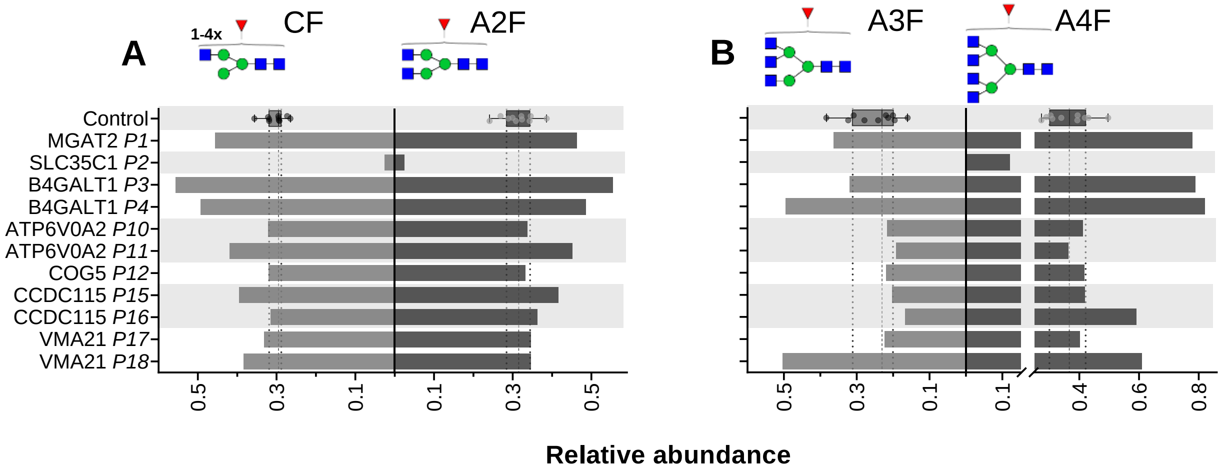

Supplementary figure S2. Total plasma N-glycome (TPNG) structures features determined for CDG patients. (**A**) Levels of complex type (left) and di-antennary (right) fucosylation and (**B**) tri-antennary (left) and tetra-antennary (right) fucosylation. The box plot shows the median (dashed line) with the interquartile range (dotted lines) for healthy controls (n = 10) whilst the bars give the individual values determined for the CDG patients.

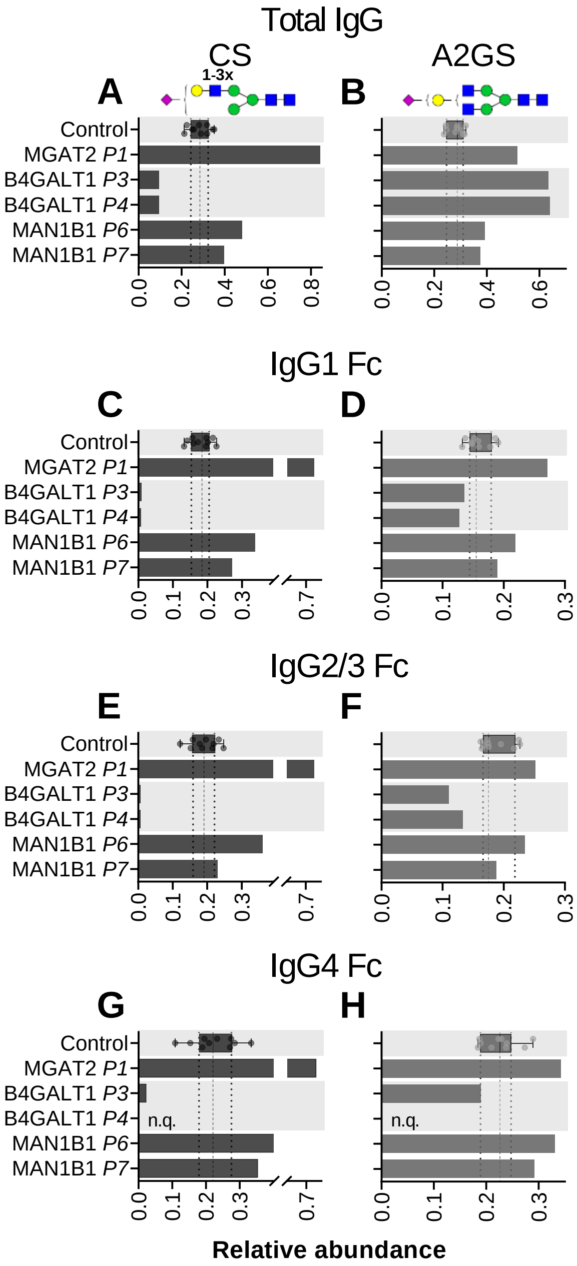

Supplementary figure S3. Relative abundances of the derived glycosylation traits sialylation (CS) and sialylation per galactose (A2GS) on total IgG (**A**, **B**) and the different IgG subclasses; IgG1 Fc (**C**, **D**), IgG2/3 Fc (**E**, **F**) and IgG4 Fc (**G**, **H**). Bars and graph are explained in Figure S2.
